# Analysis of patients’ thoughts and background factors influencing attitudes toward Deprescribing: interviews to obtain hints for highly satisfying and valid prescriptions

**DOI:** 10.1186/s40780-023-00325-7

**Published:** 2023-12-11

**Authors:** Sayaka Arai, Takahiro Ishikawa, Kenichi Arai, Takaaki Suzuki, Itsuko Ishii

**Affiliations:** 1 Division of Pharmacy, Chiba University Hospital, Chiba, Japan; 2 Geriatric Medical Center, Chiba University Hospital, Chiba, Japan; 3 Division of General Medical Science, Chiba University Graduate School of Medicine, Chiba, Japan

**Keywords:** Deprescribing, Interview, Polypharmacy, Patient satisfaction

## Abstract

**Background:**

Prescribing with high levels of medical appropriateness and patient satisfaction improves adherence. However, its appropriateness does not always match patient preference. Deprescription is important for ensuring the safety of medication therapy, but is not straightforward. Although successful deprescribing requires knowledge of patients’ thoughts on their prescriptions and factors that influence their acceptance of deprescribing, few comprehensive studies have been conducted on this topic. The aim of this study was to identify factors that influence patients’ attitudes toward deprescribing and obtain hints on how to achieve higher patient satisfaction and prescribing adequacy.

**Methods:**

A questionnaire was administered to hospitalized patients and a logistic regression analysis was conducted to examine factors that influence their attitude toward deprescribing. Individual factors affecting patients’ thoughts and wishes regarding prescribing were extracted and analysed in detail.

**Results:**

The analysis included 106 patients, of whom 40 (37.7%) wished deprescribing. Logistic regression analysis showed that “Age”, “Wish to reduce the number and types of medications”, “Satisfaction”, “Concerns about side effects”, and “Wish not to have certain medications changed” were factors influencing attitudes toward deprescribing. The results suggested that the factors were influenced by patients’ perceptions and individual patient backgrounds. There was a gap between willingness to reduce medication and to change their medications. Seventy-eight percent of all respondents indicated that they would like to reduce the number and type of pills they take if possible. However, only 44.6% of these patients indicated that they would actually like to change their medication.

**Conclusions:**

This study is the only one to comprehensively investigate prescription content, patient background, and patients’ thoughts on factors influencing attitudes toward deprescribing. This study revealed five factors that can influence inclination toward deprescribing. In addition, the results suggest that patients want to be able to feel well with fewer medications if possible. This information may be useful in determining prescriptions that have high validity and patient satisfaction. Further research is needed on the gap between willingness to reduce medications and to change medications.

## Background

Prescriptions with high medical validity and patient satisfaction improve adherence [1, 2]. However, the appropriateness of pharmacotherapy does not always match patients’ wishes. A medication may be important to a patient even if the health care provider deems it not so. Unilateral changes in prescriptions by healthcare providers can cause anxiety in patients and worsen their physical condition, making them not easy to change. This also applies to deprescribing in polypharmacy, the use of more medications than medically necessary [3]. In Japan, it is defined as a situation that increases the risk of adverse drug events, medication errors, and poor medication adherence, related to the number of medications taken rather than just taking multiple medications [4]. Increasing the number of medications results in more high-risk prescriptions, making polypharmacy more likely [5]. Recently, deprescribing has been proposed for making prescriptions more appropriate. Deprescribing means discontinuing inappropriate medications under the supervision of a healthcare professional to manage polypharmacy and improve outcomes [6]. Deprescribing is not just about reducing the types or numbers of medications, but also includes changing to more appropriate medications or dosages. Despite growing evidence for the efficacy and safety of deprescribing for polypharmacy patients [7, 8], barriers to it have been reported for patients and providers [9, 10]. Although several tools have been developed to identify patient attitudes toward deprescribing [11, 12], they do not have high predictive validity or adequately determine patient barriers and enablers [12]. Successful deprescribing requires a knowledge of factors that influence patients’ thoughts on prescription and attitudes toward deprescribing. Previous studies have not reached a consensus on associations of quantitative factors like patient age, number of medications, number of illnesses, and Potentially Inappropriate Medications (PIMs) with patient attitudes toward deprescribing [13, 14]. Qualitative studies have been conducted on patient-specific factors [15], but few have comprehensively investigated patients’ thoughts on their prescriptions and factors that influence their attitude to deprescribing. Some studies have screened for “patients with five or more medications” and “patients whose prescriptions were reviewed in advance by a medical practitioner and determined to require deprescribing”. However, it is possible that some patients not selected for deprescribing by medical staff had a latent wish for it. The aim of this study was to identify factors influencing patient’s attitudes toward deprescribing, and to provide guidance on prescribing having greater patient satisfaction and validity.

## Methods

### Preparation of the questionnaire

**Fig. 1:**
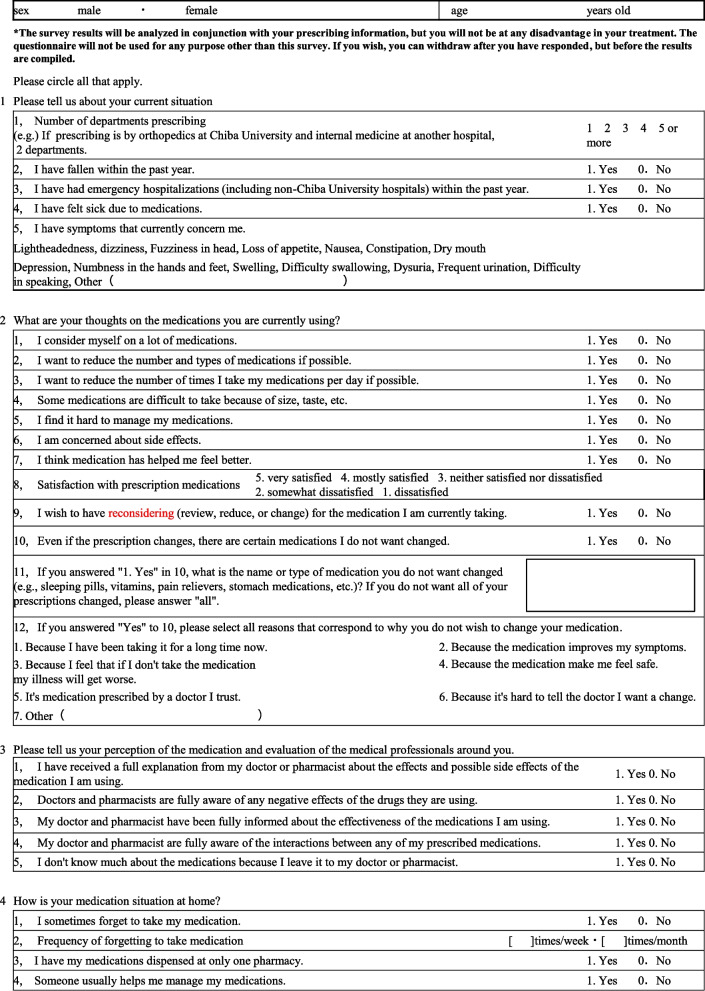
Questionnaire on medications currently used

The questionnaire was developed prior to the survey (Fig. 1). It included items (age, number of departments, number of drugs) that were related to attitudes toward deprescribing in the results of a previous study [13]. The questions also included those on items that were expected to influence attitudes toward deprescribing based on the authors’ previous experience (history of side effects, current physical condition, thoughts on prescription content, awareness of medications, and medication status). Deprescribing is not just about reducing the types or number of medications, but also includes changing to more appropriate medications or dosages. In order to obtain hints on how to make prescribing more appropriate for patients, we set items that would allow us to investigate in detail not only the number of medications but also patients’ thoughts on such aspects as number of doses per day, and dosage forms of medications. In our preliminary study, we found that some medications were more likely to be accepted by patients than others. We also discovered that a certain number of patients were reluctant to express their desire to change their prescriptions to their prescribing physicians (unpublished data). Therefore, the questionnaire was designed to ask patients specifically which medications they did not want to change and their reasons.

### Subject and data collection

The survey was initiated with a target number of 400 patients. The target number of patients was estimated, with a 5% margin of error and a 95% confidence level, based on the number of patients to whom pharmacists provided medication counseling in the previous year at our hospital (excluding emergency departments, pediatrics, and psychiatry). The commonly used formula *n* = 1.96p(1-p)/d^2^ (d: margin of error, p: response rate) was used as a reference, performing the calculation with a 5% margin of error, confidence level of 95%, and response rate of 50%. The study period was from June 2019 to March 2020. The survey was intended to last 1 year, but due to the spread of COVID-19, it actually had to be completed in 10 months. Subjects were selected from patients who were admitted to the hospital during the study period and received medication guidance from a pharmacist. Patients who could not be conversed with or were admitted to the psychiatric department were excluded, considering the influence on their decisions. Fourteen pharmacists in charge of the wards checked the condition of individual patients and determined if it was possible to request their cooperation. Thirty pharmacy interns served as interviewers. Requests for cooperation were made when the patient was present at a time that the interviewer could visit. Patients were informed of the purpose of the study in writing, and a face-to-face questionnaire was administered after obtaining their consent. In order to ensure uniformity and minimize deviations, we conducted an advanced orientation meeting for interviewers. The contents of the questionnaire, the interpretation of the questions, and how to ask patients were all thoroughly explained to them. The questionnaire was filled out by the interviewers. Prescription details were based on responses regarding medications brought to the hospital at admission. Complexity of formulations was assessed using the Medication Regimen Complexity Index (MRCI) [16].

### Statistics

Logistic regression analysis with forward selection (likelihood ratio) was used to examine factors influencing patients’ attitudes toward deprescribing. Explanatory variables were patient background factors (Age, Sex, Disease, Number of departments, Number of medications, One dose package, Falling within the past year, Emergency hospitalization within the past year, Feeling sick due to medication, Current symptoms of concern), Thoughts on prescription (Thoughts on taking many medications, Wish to reduce number and types of medications, Wish to reduce daily medication frequency, Feeling that medications are difficult to take due to taste or size, Feeling that medications are difficult to manage, Concerns about side effects, Feeling that taking a medication makes them feel better, Satisfaction, Do not want medication to be changed, Unfamiliarity with medication), and evaluation of adequacy of physician and pharmacist actions (Explanation of possible side effects, Confirmation of side effects, Confirmation of therapeutic effects, Confirmation of interactions), and home medication-related factors (Forgetting to take medications, Having family pharmacy, Having medication advisor). MRCI score was not included as an explanatory variable because it was strongly correlated with the number of medications (Spearman’s ρ = 0.978). Multiple regression analysis was used to screen for factors affecting satisfaction. The explanatory variables were factors examined in the logistic regression analysis, excluding satisfaction and attitudes toward deprescribing. Differences between elderly and non-elderly who wish deprescribing were tested using Fisher’s exact probability test. Differences in the number of medications and MRCI scores according to patient thoughts on deprescribing were tested using Mann-Whitney’s U test. Differences in the numbers of patients who did not want their medications changed were tested using Fisher’s exact probability test for each type of medication. SPSS version 24 for Windows (IBM) was used for statistical processing. The statistical significance level was set at 5%.

## Results

### Patient background

Of the 118 patients who underwent the questionnaire, 106 who responded to all questions were included in the analysis. Their backgrounds are shown in Tables 1 and 2. The median age was 70 years. The median number of medications was 8, and the median MRCI score was 29.

**Table 1:** Background of patients

	Number or Median	Percentage or range
Sex		
Male	47	44.3%
Female	59	55.7%
Age	70	31–89
<65	36	34.0%
65–74	41	38.7%
75≦	29	27.4%
Number of consultation departments	2	1–5
Number of prescribed medications	8	1–38
MRCI score	29	6–135.5
One dose package	10	9.4%
Existing diseases (ICD10)		
Neoplasms (C00-D48)	60	56.6%
Endocrine, nutritional and metabolic diseases(E00-E99)	57	53.8%
Diseases of the nervous system (G00-G99)	9	8.5%
Diseases of the eye and adnexa (H00-H59)	11	10.4%
Diseases of the circulatory system (I00-I99)	77	72.6%
Diseases of the respiratory system (J00-J99)	14	13.2%
Diseases of the digestive system (K00-K93)	17	16.0%
Diseases of the musculoskeletal system and connective tissue (M00-M99)	26	24.5%
Diseases of the genitourinary system (N00-N99)	13	12.3%

**Table 2:** Symptoms that subjects are concerned about

Symptoms	Number
Numbness in hands and feet	23
Swelling	23
Dry mouth	21
Frequent urination	21
Lightheadedness	16
Constipation	14
Depression	10
Loss of appetite	8
Dizziness	7
Dysuria	7
Fuzziness in head	3
Nausea	2
Difficulty swallowing	2
Difficulty in speaking	0
Other	13

### Factors affecting attitudes toward Deprescribing

Forty patients (37.7%) wished deprescribing. Logistic regression analysis was performed to determine factors influencing attitudes toward deprescribing among patient background, patient thoughts toward prescription, their evaluation of the health care provider’s actions, and their medication status (Table 3). Factors extracted were “Age”, “Wish to reduce the number and types of medications”, “Satisfaction”, “Concerns about side effects” and “Wish not to have certain medications changed”.

**Table 3:** Factors influencing wish for deprescribing: Results of logistic regression analysis with forward selection (likelihood ratio)

	Regression coefficient	Significant probability	Odds ratio	95% Confidence Interval
Age	0.068	0.005	1.070	1.021–1.121
Wish to reduce number and types of medications	1.570	0.032	4.807	1.147–20.147
Concerns about side effects	1.393	0.008	4.026	1.427–11.353
Satisfaction	−0.938	0.005	0.391	0.202–0.757
Wish not to have certain medications changed	1.667	0.008	5.297	1.555–18.041

χ2 model Chi-square < 0.05, Hosmer-Lemeshow 0.065, Percentage of positive identification 76.4%

#### Age

Older patients were more likely to wish deprescribing (OR: 1.07, Table 3). When patients wishing deprescription were analysed separately by age (< 65, ≥65), significant differences were found in response frequency for “Difficulty in managing medications”, “Want to reduce the number and types of medications”, “Sometimes forget to take medications”, and “Concern about side effects”. There was no significant difference in response rate for “Take a lot of medications” or “Have felt sick due to medications” (Table 4). There were no significant intergroup differences for satisfaction, number of medications, or MRCI score (Table 5).

**Table 4:** Differences between elderly and non-elderly who wish deprescribing. Patients’ thoughts

	Total		Patients who wish deprescribing (%)		*p-*value
	age<65	age≧65	age<65	age≧65	
I find it hard to manage my medications.	15	21	4 (26.7%)	13 (61.9%)	0.039^*^
I want to reduce the number of times I take my medication per day if possible.	36	57	7 (19.4%)	30 (52.6%)	0.001^**^
I consider myself on a lot of medications.	18	53	5 (27.8%)	27 (50.9%)	0.075
I sometimes forget to take my medications	21	24	5 (23.8%)	13 (54.2%)	0.038^*^
I am concerned about side effects.	19	30	6 (31.6%)	19 (63.3%)	0.030^*^
I have taken medication that made me sick.	7	17	1 (14.3%)	8 (47.1%)	0.149

Fisher’s exact test, ^*^ *p* < .05, ^**^p < .001

**Table 5:** Differences between elderly and non-elderly who wish deprescribing. Number of medications, MRCI score, Satisfaction

	Patients who wish deprescribing		*p-*value
	age<65 (*n* = 7)	age≧65 (*n* = 33)	
Number of medications (median)	8 (2–19)	9 (2–38)	0.391
MRCI score (median)	29 (7–74.5)	35 (7–135.5)	0.455
Satisfaction (median)	3 (2–4)	3 (1–4)	0.759

Mann-Whitney U-test

#### Wish to reduce the number and types of medications

“Wish to reduce the number and types of medications” was a factor influencing attitudes toward deprescribing. However, Disease, Number of departments, and Number of medications were not. Among patients who “Want to reduce the number and types of medications”, there was no difference in the median number of medications between patients who wanted deprescribing and those who did not (Table 6). Conversely, there was a significant difference in the number of medications between those who thought they were taking a lot of medications and those who did not (Table 7), indicating that the perception of the former that they were taking a large number of medications was correct. However, this was not a factor influencing attitudes toward deprescribing. In addition, although 78% of all respondents wanted to reduce the number and types of medications taken, if possible, only 44.6% of them actually wished deprescribing.

**Table 6:** Comparison of number of drugs and MRCI score according to patient thoughts on deprescribing. Patients who want to reduce the number and types of medications

	Deprescribing wished or not		*p*-value
	Wished(*n* = 37)	Not wished (*n* = 46)	
Number of medications (median)	9 (2–38)	7 (1–28)	0.392
MRCI score (median)	31 (7–135.5)	26 (6–112.5)	0.379

Mann-Whitney U-test

**Table 7:** Comparison of number of drugs and MRCI score according to patient thoughts on deprescribing. Patient thinks they are taking a lot of medications (Yes/No)

	Taking a lot of medications or not		*p*-value
	Yes(*n* = 71)	No(*n* = 35)	
Number of medications (median)	9 (3–38)	4 (1–12)	< 0.001^**^
MRCI score (median)	36 (13–135.5)	17 (6–48.5)	< 0.001^**^

Mann-Whitney U-test, ^**^p < .001

#### Satisfaction

Low satisfaction affected attitudes toward deprescribing (Table 3). Therefore, stepwise multiple regression analysis was conducted to determine factors influencing satisfaction (Table 8). Feeling well due to medication influenced satisfaction. Emergency hospitalization within the past year was a factor negatively affecting satisfaction. However, with an R^2^ of 0.202, its contribution was small.

**Table 8:** Stepwise multiple linear regression analysis for factors affecting satisfaction

	Coefficient	Standard Coefficient	Significant probability	95% Confidence Interval	VIF
(constant)	3.056		0.000	2.759–3.352	
Feeling well due to medication.	0.803	0.419	0.000	0.472–1.134	1.003
Emergency hospitalization within past year	−0.418	−0.196	0.027	−0.786-0.05	1.003

ANOVA *p* < 0.05, Adjusted R^2^ = 0.207, Durbin-Watson ratio 2.364

#### Concern about side effects

Among patients wishing deprescribing, there was no difference in the experience of side effects between those who were concerned about them and those who were not. There were also no differences in their evaluation of health care providers for explaining and confirming side effects, presence of symptoms of concern, or self-evaluation of knowledge of medications (data not shown).

#### Medication patients did not want changed

Tables 9 and 10 shows a breakdown of medications respondents did not want changed. Antihypertensives, analgesics, and sleeping pills accounted for 60% of them (Table 9). Not wanting to change these medications was significantly more likely than for dyslipidaemia medications. The most common reason given for not wanting a change was “Taking medication for a long time”. Only one respondent found it difficult to tell the doctor they wanted a change (Table 10).

**Table 9:** Medications that patients do not want changed. Breakdown of medications

	Number of patients who do not want their medications changed (*n* = 24)	Number of patients prescribed medications (*n* = 106)	Percentage (%)	Comparison with dyslipidemia drugs (*p*-value)
Antihypertensives	9	74	12.2	0.008^**^
Analgesics	3	30	10	0.049^*^
Sleep medicines	3	17	17.6	0.014^*^
Digestive agents	2	62	3.23	0.304
Chinese medicine	1	18	5.56	0.265
Anti-thrombotic drugs	3	44	6.82	0.099
Diabetes medicines	1	23	4.35	0.315
Anti-arrhythmic drugs	1	12	8.33	0.194
Hyperuricemia agents	1	19	5.26	0.275
Lipid disorder drugs	0	50	0	–

Fisher’s exact test, ^*^ *p* < .05, ^**^p < .001

**Table 10:** Medications that patients do not want changed. Reason

	Number of patients (*n* = 24)	Percentage(%)
Because I have been taking it for a long time now	12	50.0
Because the medication improves the symptoms	9	37.5
Because I feel like if I don’t take the medicine, my illness will get worse	6	25.0
Because the medication makes me feel safe	8	33.3
It’s medication prescribed by a doctor I trust	7	29.2
Because it’s hard to tell the doctor I want a change	1	4.2

## Discussion

### Novelty of this study

Various studies have been conducted on patient attitudes toward deprescribing. However, few have investigated patients’ thoughts and wishes regarding prescribing. This study is the only one to comprehensively investigate prescription content, patient background, and patients’ thoughts on factors influencing attitudes toward deprescribing.

### Five factors influencing attitudes toward Deprescribing

The five factors extracted in the logistic regression analysis are discussed below.

#### Age

A systematic review of attitudes toward deprescribing indicated that many older adults wish to reduce medications if clinicians agree [12]. Previous studies in Japan have reported that patients’ willingness to reduce medication increases with age [13]. In our results, the wish for deprescribing was also correlated with older age. Why does the wish for deprescribing increase with increasing age? With aging, diseases increases and the number of medications increases [17, 18]. Some studies have found that the number of medications affects adherence [19], suggesting that as the number of medications increases, patients experience difficulty in managing medications and become more positive about deprescribing. In our study, there were no significant differences in the number of medications or MRCI scores between elderly and non-elderly patients who requested deprescribing. However, significantly more elderly respondents selected “difficulty in managing medications,” “want to reduce the number and types of medications,” and “concern about side effects”. Even if the number of medications and the complexity of prescriptions are the same, the elderly may be less able to manage their medications than non-elderly patients, and more concerned about side effects due to a perceived decline in physical strength and physiological function, which may increase motivation for deprescribing.

#### Wish to reduce the number and types of medications

“Wish to reduce the number and types of medications” was a factor influencing attitudes toward deprescribing, while the number of medications was not. A meta-analysis reporting that patients’ and caregivers’ willingness to reduce medication was not affected by the number of medications [12] seems to support our results. Patients’ perception that they were “taking a lot of medications” was correct, but this did not affect the wish for deprescribing, suggesting that patients were aware of taking many medications but deemed deprescribing unnecessary if they felt that all were necessary.

#### Satisfaction with prescribing

In our study, a positive factor affecting satisfaction was the feeling that medication helped patients feel better while a negative one was experiencing emergency hospitalization within the past year, both influencing patient evaluation of medication efficacy. Medications they did not want changed may have been those felt to readily improve symptoms, such as antihypertensives and analgesics. However, the goodness of fit in multiple regression analysis was low (Table 8). Thus, these 2 factors are insufficient for explaining the satisfaction level. It has been reported that the efficacy, side-effect profile, and regimen of a medication can affect patient satisfaction [20–22] and were also factors considered in this study. Factors influencing satisfaction may differ from person to person.

#### Concern about side effects

We assumed that “Concern about side effects” indicated patient anxiety about continuing to take a medication. However, there was no difference between patients who wanted deprescribing and those who did not regarding the experience of side effects, current symptoms of concern, and evaluation of the health care provider’s explanation and confirmations. For patients who wished deprescribing, there was no difference between those who were concerned about side effects and those who were not (data not shown). Therefore, it is necessary to determine points of concern for each patient and make efforts to alleviate them by skillfully devising the explanation content.

#### Deprescribing preference by prescription medication type

It seems contradictory at first glance that having a medication that one does not want changed influences one’s attitude toward deprescribing. However, the response “There are medications that I do not want changed” can be seen as indicating a willingness to be actively involved in medication treatment. However, with a good understanding of their medications, patients may want to see them changed to those that are more effective in improving their symptoms. Respondents did not want to have antihypertensive drugs, sleeping pills, or painkillers changed because they likely benefitted from them. Dyslipidemia drugs, which were used for comparison, are often prescribed for the prevention of cardiovascular events, but it is difficult for patients to understand the necessity of taking them. For medications whose evaluation of efficacy is highly subjective, patients may not want them changed for fear of their condition worsening or reduced efficacy. The most common reason given for not wanting a change of “Because I have been taking it for a long time” supports this. Patients may wish to have medications whose efficacy is difficult to perceive reviewed. The results of this survey may reflect these mindsets.

### How to achieve patients’ wishes for prescribing

Overall, the above results suggest that patients want to feel well with fewer medications. In other words, patients want prescriptions that use the least number of medications, minimize side effects, and provide a stable therapeutic effect. They also suggest that the five factors influencing attitudes toward deprescribing depend on individual patient perception and background factors. Our findings indicate the necessity of close communication with patients and determining their needs to ensure that prescriptions are tailored to individual patients and achieve a high degree of satisfaction. In addition, we found that approximately 80% of the analysed subjects had a latent wish to reduce the number and types of medications and this was independent of the number of medications prescribed. This suggests that medical providers should proactively make appropriate prescribing suggestions regardless of the number of medications. Pharmacist medication reviews alone or in combination with adherence reviews have been reported to improve patient satisfaction equally [23]. Regular medication reviews and making recommendations for appropriate prescribing based on patient understanding may help address potential patient needs and increase satisfaction with prescribing. In light of these considerations, healthcare providers should keep the following practices in mind: 1. Listen more attentively to the patient’s thoughts through closer communication. 2. Regardless of the number of medications a patient is taking, proactively suggest changes to prescriptions that are commensurate with the patient’s ability to manage their medications, or appropriate medication reductions or prescription changes that improve the quality of medication therapy.

In Japanese clinical practice, patients are commonly selected for deprescribing based on a certain number of medications; e.g., five or more, so that it can be performed efficiently with limited manpower. However, this intervention is from the medical professional’s perspective. Our results showed that the perception of being on multiple medications and attitudes toward deprescribing varied from patient to patient. Including the patient’s perspective, it seems suboptimal to use number of medications as a basis for intervention. Focusing on patients’ medication management skills and thoughts on prescribing would be more in line with their wishes. A disadvantage, however, would be the time taken to gather information. A future issue will be how to efficiently conduct prescribing interventions from the patient’s standpoint. Recently, Kim et al. [24] reported that clinicians were able to elicit individual barriers and enablers to deprescribing from the patient’s perspective using a semi structured interview conversation tool. We hope that the development of such practical tools will increase in the future, in which the five factors obtained in the survey – “Age”, “Wish to reduce the number and types of medications”, “Satisfaction”, “Concerns about side effects,” and “Wish not to have certain medications changed”- are likely to be important factors.

### Gap between willingness to reduce medications and to change medications

This study found that although about 80% of patients wanted to reduce the number and types of medications, more than half of them did not actually wish deprescribing. A high percentage of patients were reported to be willing to stop one or more medications if their doctor said it was possible [11, 12, 25], and Turner et al. reported that 86% of study participants indicated a willingness to reduce their medications in a preliminary survey, but only 41% were able to reduce them [26]. Our results suggest that although patients wanted to reduce their medications, they were hesitant to change prescriptions because of concerns about symptoms worsening or changes in their condition, or perhaps they wanted to reduce their medications but had given up the idea feeling that all medications prescribed by their physicians were necessary for treatment. However, since examination of this gap was not a major objective, a detailed analysis was not performed, so further research is needed in the future.

### Strengths and weaknesses

In most related studies conducted to date, patients were enrolled through screening according to the use of multiple medications or the necessity of prescribing interventions. Our study also considered age, number of medications, and prescription content, which we believe allowed us to determine prescribing needs for all patients. However, there are several limitations. First, the number of patients included in the analysis was not so large. The results may have been a little less reliable than we had anticipated during the planning phase, since we were not able to collect the target number of patients. However, since the sample size required for a survey with a 10% margin of error and a 95% confidence level is generally less than 100 persons, the results of this study are considered to have a certain degree of acceptability and reliability. Second, the questionnaire was administered to patients whom the pharmacist in charge of the hospital ward determined to be able to request cooperation in view of the patient’s condition, i.e., patients in relatively good condition, making it possible that many patients proactive toward medication treatment were selected. Since patients were informed of the survey’ purpose and then their consent was obtained, giving consent to the survey itself may also have served to screen for patients who were proactive toward drug treatment and interested in deprescribing. In addition, since the questionnaire was administered face-to-face, it is possible that it did not elicit accurate responses to questions requiring an evaluation of healthcare providers. Furthermore, 30 interviewers were involved in this study. In general, the large number of interviewers can lead to variations or flaws in the data. To ensure data consistency and minimize deviations, we conducted an advanced orientation meeting for interviewers. Moreover, we minimized this concern by using a multiple-choice questionnaire during the interviews.

## Conclusions

Five factors were associated with attitudes toward deprescribing: “Age” “Wish to reduce the number and types of medications” “Satisfaction”, “Concerns about side effects”, and “Wish not to have certain medications changed”. In addition, the results suggest that patients want to be able to feel well with fewer medications if possible. This information may be useful in determining prescriptions that have high validity and patient satisfaction. Further research is needed on the gap between willingness to reduce medications and to change medications.

## Data Availability

The data that support the findings of this study are available from the authors but restrictions apply to the availability of these data, which were used under license from Chiba University Hospital for the current study, and so are not publicly available.

## References

[1] Alkatheri AA, Albekairy AM, Jarab A (2016). Medication adherence and treatment satisfaction among renal transplant recipients. Ann Transplant..

[2] Jacobs JM, Pensak NA, Sporn NJ (2017). Treatment satisfaction and adherence to Oral chemotherapy in patients with Cancer. J Oncol Pract..

[3] Tjia J, Velten SJ, Parsons C (2013). Studies to reduce unnecessary medication use in frail older adults: a systematic review. Drugs Aging..

[4] Ministry of Health, Labour and Welfare. “Koureisha no iyakuhin tekiseishiyou no shishin (sousyuhen)” [Guidelines for the Proper Use of Medicines for the Elderly (compilation)]. 2018. https://www.mhlw.go.jp/content/11121000/kourei-tekisei_web.pdf, Accessed 18.6.2023. (in Japanese).

[5] Arai S, Ishikawa T, Kato H (2019). Multidrug use positively correlates with high-risk prescriptions in the Japanese elderly: a longitudinal study. J Pharm Health Care Sci..

[6] Reeve E, Gnjidic D, Long J (2015). A systematic review of the emerging definition of 'deprescribing' with network analysis: implications for future research and clinical practice. Br J Clin Pharmacol..

[7] Page AT, Clifford RM, Potter K (2016). The feasibility and effect of deprescribing in older adults on mortality and health: a systematic review and meta-analysis. Br J Clin Pharmacol..

[8] Ibrahim K, Cox NJ, Stevenson JM (2021). A systematic review of the evidence for deprescribing interventions among older people living with frailty. BMC Geriatr..

[9] Reeve E, Tos J, Hendrix I, Shakib S (2013). Patient barriers to and enablers of deprescribing: a systematic review. Drugs Aging..

[10] Gillespie RJ, Harrison L, Mullan J (2018). Deprescribing medications for older adults in the primary care context: a mixed studies review. Health Sci Rep..

[11] Weir KR, Ailabouni NJ, Schneider CR (2022). Consumer attitudes towards Deprescribing: a systematic review and Meta-analysis. J Gerontol A Biol Sci Med Sci..

[12] Chock YL, Wee YL, Gan SL (2021). How willing are patients or their caregivers to Deprescribe: a systematic review and Meta-analysis. J Gen Intern Med..

[13] Aoki T, Yamamoto Y, Ikenoue T (2019). Factors associated with patient preferences towards deprescribing: a survey of adult patients on prescribed medications. Int J Clin Pharm..

[14] Komagamine J, Sugawara K, Hagane K (2018). Characteristics of elderly patients with polypharmacy who refuse to participate in an in-hospital deprescribing intervention: a retrospective cross-sectional study. BMC Geriatr..

[15] Weir K, Nickel B, Naganathan V (2018). Decision-making preferences and Deprescribing: perspectives of older adults and companions about their medicines. J Gerontol B Psychol Sci Soc Sci..

[16] George J, Phun YT, Bailey MJ (2004). Development and validation of the medication regimen complexity index. Ann Pharmacother..

[17] Ornstein SM, Nietert PJ, Jenkins RG (2013). The prevalence of chronic diseases and multimorbidity in primary care practice: a PPRNet report. J Am Board Fam Med..

[18] Khezrian M, McNeil CJ, Murray AD (2020). An overview of prevalence, determinants and health outcomes of polypharmacy. Ther Adv Drug Saf..

[19] Franchi C, Ardoino I, Ludergnani M (2021). Medication adherence in community-dwelling older people exposed to chronic polypharmacy. J Epidemiol Community Health..

[20] Itou H (2011). “Huminshou kanja no manzokudo wo takameru suimin yaku chiryou toha – daikibo internet kanja chousa no kekka kara” [What Sleep Medication Treatment Increases Insomnia Patient Satisfaction? From One Large Internet Patient Survey]. J New Rem & Clin..

[21] Volpicelli Leonard K, Robertson C, Bhowmick A (2020). Perceived treatment satisfaction and effectiveness facilitators among patients with chronic health conditions: a self-reported survey. Interact J Med Res..

[22] Flood EM, Bell KF, de la Cruz MC (2017). Patient preferences for diabetes treatment attributes and drug classes. Curr Med Res Opin..

[23] Bou Malham C, El Khatib S, Cestac P (2021). Impact of pharmacist-led interventions on patient care in ambulatory care settings: a systematic review. Int J Clin Pract..

[24] Kim JL, Lewallen KM, Hollingsworth EK, et al. Patient-reported barriers and enablers to deprescribing recommendations during a clinical trial. Gerontologist. 2022:gnac100. 10.1093/geront/gnac100. 10.1093/geront/gnac100 PMC10028229 35881109

[25] Reeve E, Wolff JL, Skehan M (2018). Assessment of attitudes toward Deprescribing in older Medicare beneficiaries in the United States. JAMA Intern Med..

[26] Turner JP, Martin P, Zhang YZ (2020). Patients beliefs and attitudes towards deprescribing: can deprescribing success be predicted?. Res Social Adm Pharm..

